# Responding to the burden of dementia in Africa: Does cultural myth and perception of dementia impact clinical professionals' attitude, knowledge and experiences of dementia? Finding from qualitative interviews of newly migrated Nigerian clinical professionals in the UK

**DOI:** 10.1002/alz70860_102967

**Published:** 2025-12-23

**Authors:** Emmanuel Sunday Nwofe, Centre for Applied Dementia Studies

**Affiliations:** ^1^ University of Bradford, Bradford, West Yorkshire, United Kingdom

## Abstract

**Background:**

The burden of dementia in Africa is increasing due to a rapidly growing ageing population, a broken social welfare system, and a lack of awareness about the condition, which is often influenced by cultural and religious beliefs. In many African languages and cultures, there is no specific word for dementia. Instead of benefiting from early interventions and clinical management, older individuals are expected to experience a decline in cognitive functions as a normal part of ageing, frequently associating this decline with witchcraft or sorcery. As a result, many people deny that dementia is a medical condition, and those displaying symptoms are often isolated and left in traditional (unorthodox) treatment centres. Clinical professionals play a crucial role in raising awareness about dementia and its risk factors in order to dispel misconceptions surrounding the condition.

**Method:**

Fifteen clinical professionals, including nurses and physicians who recently migrated to the UK, were interviewed using semi‐structured interview guidelines. The interviews lasted 45‐60 minutes and were recorded and transcribed verbatim via Microsoft Teams. Data analysis was completed on NVivo 12 Pro software following Braun and Clark's Thematic Analytical guidelines

**Result:**

The study found that dementia is “more of a textbook knowledge” for clinical practitioners in Nigeria, with practice often influenced by cultural perceptions and beliefs of dementia as a normal part of ageing process. Four main themes were categorised from the interview transcripts, including i) cultural influence and awareness, ii) clinical training and case experiences, iii) limited expertise and resources and iv) government priorities in mental health conditions.

**Conclusion:**

Raising awareness of dementia in Africa is the first significant step in tackling the burden of the condition on the continent**,** including the involvement of institutions, organisations and policies. Without requisite training and experiences of clinical professionals in dementia care and available resources to diagnose dementia, the attitude and knowledge of dementia among the lay populace would always be influenced by cultural stigma and misconception.

